# AI-driven strategies for advancing corneal cell therapy: a promising frontier

**DOI:** 10.3389/fmed.2025.1563891

**Published:** 2025-10-02

**Authors:** Mahsa Fallah Tafti, Masoud Khorrami-Nejad, Masoud Arabfard, Mohsen Ghiasi, Fatemeh Afkhamizadeh, Khosrow Jadidi, Hossein Aghamollaei

**Affiliations:** 1 Vision Health Research Center, Semnan University of Medical Science, Semnan, Iran; 2 School of Rehabilitation, Tehran University of Medical Sciences, Tehran, Iran; 3 Optical Techniques Department, College of Health and Medical Techniques, Al-Mustaqbal University, Babylon, Iraq; 4 Artificial Intelligence in Health Research Center, Biomedicine Technologies Institute, Baqiyatallah University of Medical Sciences, Tehran, Iran; 5 Cardiovascular Research Center, Rajaie Cardiovascular Institute, Tehran, Iran; 6 Chemical Injuries Research Center, Systems Biology and Poisonings Institute, Baqiyatallah University of Medical Sciences, Tehran, Iran

**Keywords:** artificial intelligence, cornea, cell therapy, regenerative medicine, personalized medicine

## Abstract

Cell-based therapies offer an alternative to corneal transplantation for the management of corneal diseases. However, these approaches require a deeper understanding of the principles of cell therapy, and the ability to predict and diagnose outcomes pre- and post-operatively is highly desirable. Recently, the development of innovative techniques that leverage predefined data from multiple cohorts with corneal diseases has received considerable attention. Approaches using artificial intelligence (AI) can address major concerns in corneal cell therapy, including the identification of novel biomarkers, improvements in cell delivery processes, and the acceleration of personalized treatments. This review summarizes real-world examples of AI applications from preclinical through clinical studies, with a focus on corneal cell-based therapies.

## Graphical Abstract

**Figure:**
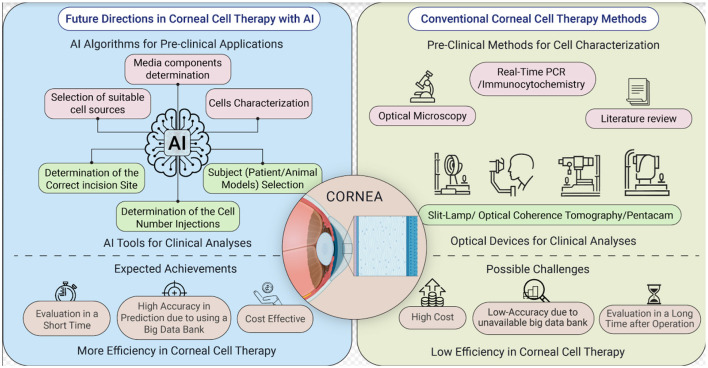
A schematic illustrating the evolution of corneal cell therapy from conventional methods to AI-enabled approaches. Conventional cell therapies may have lower accuracy due to missing prerequisites, such as large, high-quality datasets. By contrast, AI is expected to improve corneal cell therapy. AI-based methods, which offer advantages in accuracy, speed, and quality, hold strong promise for corneal cell therapy applications.

## 1 Introduction

The cornea is a transparent tissue with a protective responsibility for the eye (1, 2). Composed of the epithelium, stroma, and endothelium, it plays a critical role in light refraction and vision (3). According to the World Health Organization (WHO), corneal blindness is the fourth leading cause of blindness globally (4). Corneal transplantation represents a potential therapeutic intervention to manage corneal diseases and restore vision. However, this approach faces significant challenges, including donor shortage and the risk of graft rejection (5, 6). As an alternative, cell-based therapies have emerged as promising strategies for treating these conditions. Stem cells, characterized by their undifferentiated state and capacity for both self-renewal and differentiation, are central to this approach. Consequently, regenerative medicine has garnered significant attention as a potential therapeutic avenue for corneal regeneration (7, 8). This field is advancing rapidly within healthcare, offering promising solutions for repairing and restoring specific tissues, particularly in cases where the body's innate regenerative capacity is insufficient to facilitate complete healing. Cell-based therapy was developed for various corneal layers like endothelium, stroma, and epithelium in pre-clinical and clinical studies (9–13). However, conventional cell therapies face limitations such as difficulties in scaling up, high costs, and batch-to-batch variability.

They are often time-intensive and may lead to unwanted outcomes due to human error. Additional challenges include appropriate case selection, accurate cell dosing and characterization, determination of the target site, management of potential complications such as allograft rejection, edema, *in situ* infection, and neovascularization, as well as the prediction of post-operative recovery time. Therefore, it is necessary to consider effective strategies to overcome these limitations and advance corneal cell therapy.

Artificial intelligence (AI), a burgeoning discipline within the realms of computer science and engineering, has exhibited promising applications for various medical domains. During a symposium held at Dartmouth College in 1956, John McCarthy, a computer scientist, provided a formal definition for the concept of “Artificial Intelligence” (AI) (14). AI possesses the capacity to extract comprehensive and detailed information from diverse sources, including genomics, transcriptomics, proteomics, digital pathological images, and other datasets (15). This ability empowers clinicians to acquire a holistic and integrated comprehension of the subject under investigation. Additionally, AI has the potential to identify unknown biomarkers through data analysis, thereby facilitating the screening, detection, diagnosis, treatment, and prognosis prediction of various diseases. This potential allows for the provision of personalized treatment to individual patients, ultimately leading to improved clinical outcomes. Predictions in clinical trials can benefit from additional impartial assessments using cutting-edge computational tools, such as machine learning-based patient classification. Researchers constantly test hypotheses about how AI and other cutting-edge technologies can influence the future of corneal regenerative medicine (16, 17).

AI has the potential to significantly improve global healthcare in areas like assessing stem cell viability, biosafety, and selecting suitable patients (18, 19). Although AI's full capability has not yet been realized, the field of ophthalmology is already making significant strides in using the technology to improve therapeutic outcomes.

**Figure 1:**
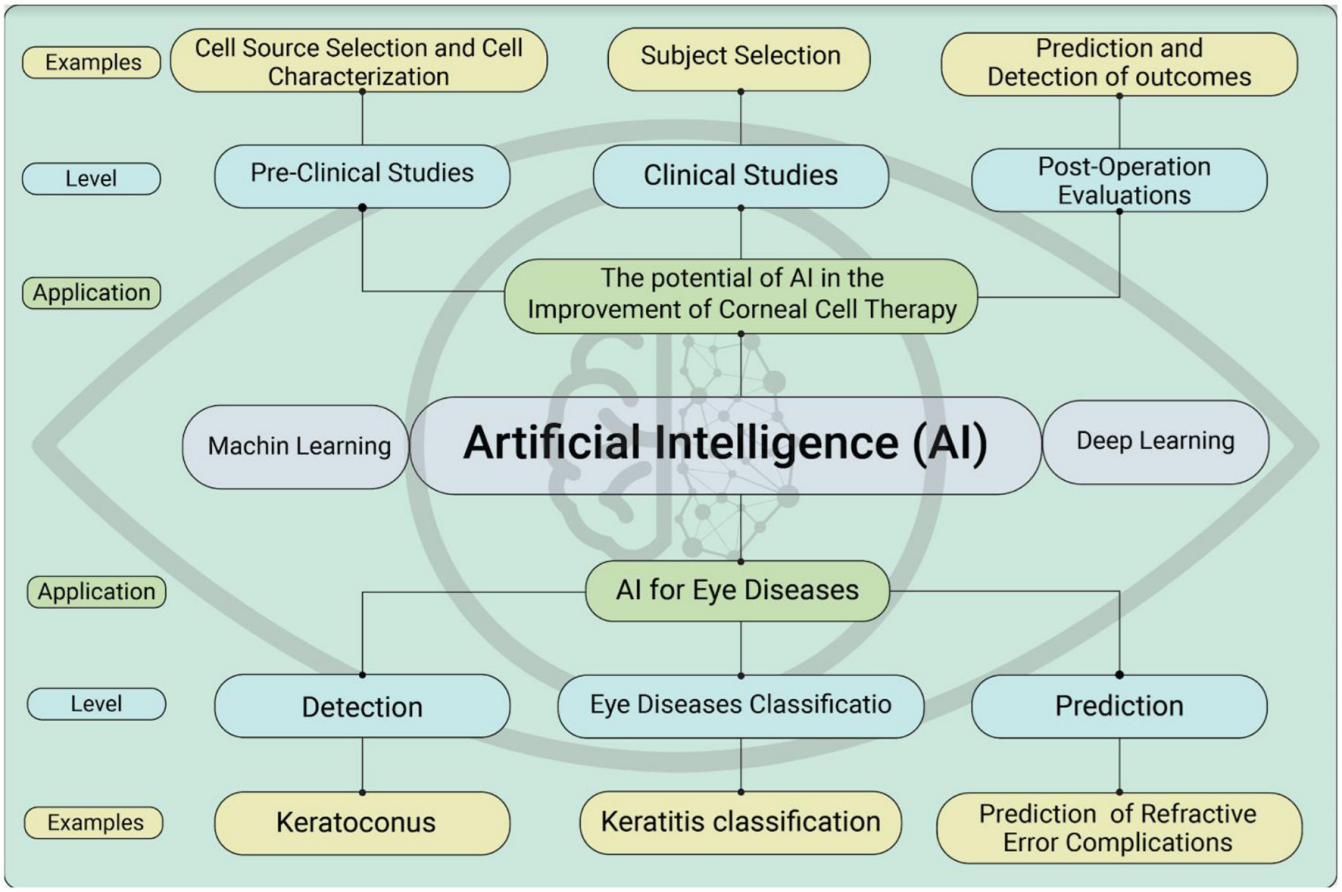
Applications of artificial intelligence for predicting and diagnosing eye diseases and for improving cell therapy in preclinical and clinical settings.

AI approaches to optimize corneal cell therapies and improve corneal regeneration would be highly beneficial. AI tools are also suggested for use in pre-clinical studies, including media component selection, cell characterization, and detection of cellular infections. Notably, AI approaches for corneal cell therapy in clinical studies include patient selection, determination of cell dosage and target site, as well as the anticipation of post-operative recovery time. In this paper, we explore the transformative potential of AI in advancing corneal cell therapy and address key challenges such as selecting the appropriate cell source, optimizing cell dose, improving case selection, and managing intraoperative and post-operative complications. Given AI's capacity to predict surgical outcomes, our aim is to bridge the gap between AI research and clinical practice by emphasizing applications across both preclinical and clinical stages of corneal cell therapy. We also highlight the role of AI in predicting and diagnosing outcomes using real-world examples. Ultimately, we seek to inform strategies for managing corneal diseases. Through this comprehensive review, we underscore the importance of integrating AI technologies into corneal cell-based treatments and outline future research and implementation pathways (Figure 1).

## 2 AI applications for detection and prognosis of corneal diseases

The cornea and lens are considered to be the primary refractive components of the ocular system. Potential consequences of structural damage include the possibility of vision problems and blindness (20). The high incidence of myopia in East and Southeast Asian countries is attributed to extensive educational practices and widespread digital learning platforms (21). AI facilitates the acquisition of knowledge, logical thinking, and goal attainment by computer systems and minimizes the reliance on human intervention (22, 23). The purpose of AI in ophthalmology is to improve the field's understanding and investigation. This development is supported by the widespread adoption of machine learning and deep learning (22, 24, 25). AI for ocular applications like detection of keratoconus (26–31), microbial keratitis (32–34), dry eye disease (35), pterygium (36–38), keratoconus management (39), and refractive error prediction (40) has been employed. Other applications of AI approaches include the prediction of keratoplasty outcomes (41, 42), the determination of fungal and bacterial keratitis (43), corneal neovascularization detection (44), and assessment of Fuchs endothelial corneal dystrophy (FECD) (45).

The process of diagnosing and planning treatment in the field of ophthalmology relies significantly on clinical examination and advanced imaging techniques. Slit-lamp photography, optical coherence tomography (OCT), tomography, and *in vivo* confocal microscopy (IVCM) are all commonly used to diagnose and monitor anterior segment diseases. However, it is important to understand that these procedures can be time-intensive and may also be susceptible to potential human errors (46). The current machine learning approach successfully categorized corneal data captured with a Pentacam to identify keratoconus (47). Patients undergoing refractive surgery can be reliably classified into stable cases and clinical ectasia using a random forest (RF) model trained on Pentacam measurement data (48). A previous study forecasted the occurrence of various ocular disorders for training and validating AI-based machine learning methodologies (49). Various images depicting eye diseases such as diabetic macular edema (DME) and choroidal neovascularization (CNV), glaucoma, normal conditions, and cataracts have been employed. The study employed various deep transfer learning approaches, including basic convolutional neural networks (CNN), deep CNN, AlexNet 2, Xception, Inception V3, ResNet 50, and DenseNet121. The obtained simulation results confirmed that the ResNet50 model achieved a validation accuracy of 98.9%, surpassing the performance of all alternative methodologies. Furthermore, the Xception model exhibited commendable performance, attaining an accurate rate of 98.4%. Despite the implementation of standardized pre-operative screening, the identification of eyes at risk of developing procedure-induced ectasia remains a considerable challenge. Furthermore, there is a development of AI platforms to screen individuals with a heightened risk of post-LASIK ectasia and vision impairment. Yoo et al. developed a machine learning platform to aid the clinical decision-making for refractive surgery (50). Another investigation was conducted to develop deep learning models to predict the post-operative outcomes of SMILE surgery, such as visual acuity and intraoperative complications, based on surgical videos or images (51). In a recent review, the integration of deep learning with advanced imaging and liquid biopsy biomarkers is highlighted as a transformative approach for understanding ocular aging and its implications for systemic health (52).

## 3 AI Applications for pre-clinical corneal cell therapy

Mathematical modeling can facilitate the identification of cellular characteristics and their microenvironments by examining the cell morphology and healthy cells (53, 54). AI-driven models and constructive algorithms offer robust solutions for gaining a more profound comprehension of these mechanisms. These models can also automate the development of regenerative medicines, thereby reducing the occurrence of human errors (19) (Table 1).

**Table 1:** Some of the AI responsibilities in pre-clinical studies.

**AI task**	**AI model**	**References**
Cell source selection	Convolutional neural networks (CNNs)	(60)
Optimization of media component	Response surface methodology (RSM), genetic algorithm (GA), and radial basis function (RBF)	(68)
Number of live cells	Phase imaging with computational specificity (PICS)	(71)
Cell morphology	Convolutional neural networks and transformers	(73)
Cell confluency and contamination	Convolutional neural networks and transformers	(73)

### 3.1 AI for determination of suitable cell source and tissue

Various cells, as well as synthetic or natural tissues, are used in corneal tissue engineering (55–57). Accuracy in selecting suitable cell sources is vital to achieving desirable outcomes in cell therapy studies. Automated cell culture platforms enhance technical accuracy, replicability, and efficiency. These platforms also integrate modern imaging techniques and analysis tools for pre-clinical applications (58). Furthermore, AI approaches can be employed to identify and predict the process of generating induced pluripotent stem cells (iPSCs) through cellular reprogramming. This enables the precise forecasting of iPSC generation and subsequent differentiation (59). For instance, CNNs can play a significant role in image identification and use deep learning to correct data attributes. By analyzing cellular changes in morphology and texture, it can reliably identify individual cells. Thus, CNNs have the potential to pave the way for a new field of deep learning tasks geared toward addressing diverse issues in stem cell research (60). CNNs may be applicable for the selection of an appropriate cell source for corneal cellular studies. For example, choosing a suitable source of stem cells based on their ability to differentiate into corneal cells will be an excellent AI application. Therefore, ensuring the selection of stem cells under *in vitro* conditions can be very effective in future investigations. Moreover, AI algorithms for determining specific features like cellular morphology, molecular ligands, and membrane receptors of corneal cells for both *in vitro* and clinical applications would be beneficial. In addition, the detection of key differentiation pathways in stem cells toward specific corneal cells in a short timeframe by AI approaches will be beneficial for corneal regeneration.

Preparing suitable donor tissue is another requirement for successful keratoplasty. An AI-based program, P06-A143, was developed to assist in diagnosing cornea guttata in donor corneas at the eye bank (61). This tool may help reduce keratoplasty complications related to donor tissue selection.

### 3.2 AI for determination of cell culture media components

Until now, many studies for culturing corneal cells under a pre-defined condition have been conducted. However, the accurate determination of the media components is important (62–67). Nikkhah et al. reported a reduced-serum culture media formulation, including insulin-like growth factor I (IGF-I), Fibroblast growth factor (FGF), transforming growth factor (TGF), platelet-derived growth factor (PDGF), selenium, ascorbic acid, and serum as independent variables for cultivated meat using response surface methodology (RSM) (68). The culture medium formulation was optimized using a genetic algorithm (GA), and radial basis function (RBF) neural networks were used for the prediction of dependent variables. Finally, a multi-objective optimization algorithm was utilized to calculate the ideal quantities of the independent variables with the three RBF neural network prediction models serving as inputs. This study's proposed RSM+ RBF + GA framework could be used to sustainably improve the production of serum-free media by determining the mix of media elements. It aims to strike a balance between yield, environmental impact, and cost, particularly for different cultured meat cell lines.

Machine learning can optimize culture media formulations and proliferation protocols by analyzing historical data from successful batches. This approach is feasible through a focus on modeling (69). AI-based robotics can ensure stable conditions for cell growth (such as temperature, pH, and nutrient supply) and reduce variability in the production of corneal cells (including limbal epithelial cells and corneal endothelial cells) (70).

Therefore, cell-based investigations aim to determine and predict culture media components to save time and cost, while attaining desired outcomes. In addition to the importance of media components for culturing cells, determining the media ingredients for stem cell differentiation into specific cells would be very effective, and AI algorithms can be used to optimize these conditions.

### 3.3 AI for determination of live cell numbers

**Figure 2:**
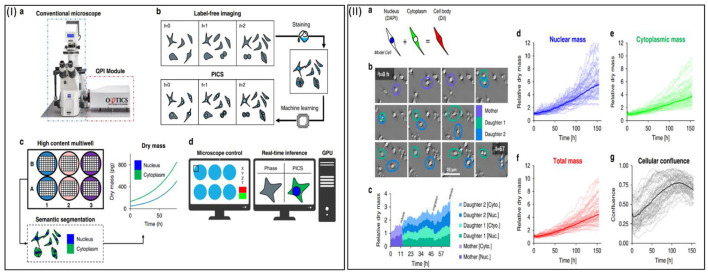
**(I)** The PICS method enables label-free measurement of cellular dry mass in specific compartments. **(II)** PICS allows monitoring of variations in the dry mass of cellular compartments. This figure is modified and reprinted from reference (71).

As mentioned earlier, it is important to take the phenotypic properties and an adequate quantity of *in vitro* cultured cells by considering their functions. Fluorescence microscopy has emerged as an indispensable imaging technique in the field of cell biology, owing to its remarkable specificity. Nevertheless, fluorescence microscopy is still constrained by factors such as photobleaching, phototoxicity, and associated artifacts. In a prior study, the ability of AI to convert one type of contrast into a different form is shown (Figure 2) (71). The authors introduced a novel technique called phase imaging with computational specificity (PICS), which integrates quantitative phase imaging and AI to offer precise details regarding unlabeled viable cells. This imaging system facilitates automated training, with the inference process integrated into the acquisition software and operating in real time. The fluorescence maps were subsequently utilized to analyze the quantitative phase imaging (QPI) data. In this study, the PICS implementation provided a flexible quantitative method for the continuous and simultaneous monitoring of specific cellular components that require prolonged label-free imaging. This AI-based approach would be applicable for counting live corneal cells in an adhesive culture plate or substrate.

### 3.4 AI for determination of infected cells

For clinical studies, considering the safety characteristics of grown cells and the other requirements for the cell culture due to the corneal-privileged system is important. The AI algorithms for prescreening and pre-classifying image data are considered to increase productivity and precise diagnosis. Expert clinical microbiologists provide crucial training in image-based infectious disease diagnoses through human interpretation. Despite the extensive time and effort required for training, validation, and implementation, AI-based diagnosis is practically cost-effective, and the majority of microbiology laboratories already have the hardware necessary to perform image analysis (72).

AI-based imaging can analyze cell morphology, confluence, and contamination in real time, ensuring that only high-quality cells proceed to treatment. This approach can be implemented using concepts such as machine vision (73).

## 4 Role of AI in clinical corneal cell therapy

Understanding early complications and outcomes within the first few days after corneal surgery is crucial for physicians. AI algorithms have the potential to help healthcare staff at different levels of this process (Table 2).

**Table 2:** AI-based approaches in clinical studies.

**AI task**	**AI-based model**	**References**
Subject selection	Support vector machines (SVMs), Random forests (RF), Artificial neural networks (ANNs), AdaBoost, LASSO	(50)
Predict the nomograms for SMILE	AdaBoost	(82)
Cell properties identification	*In-vivo* confocal microscopy (IVCM)	(83)
Graft detachment	Deep neural network (VGG19)	(42)
Infections	Deep learning models (ResNet50, ResNeXt50, DenseNet121, SE-ResNet50, EfficientNets B0, B1, B2, and B3)	(32)
Corneal edema	Deep learning-assisted Second Harmonic Generation Microscopy (SHG) imaging	(87)
Neovascularization	Optical coherence microscopy (OCT)-based machine learning	(89)
Detection of changed cell morphology	Morphogo system	(92)
Biomarkers detection	Machine learning algorithms	(117)
Detection of cell distance	Hidden Markov Model and Neural Networks	
Corneal curvature	AI-based approaches	(104, 105)

### 4.1 AI to select suitable subjects

**Figure 3:**
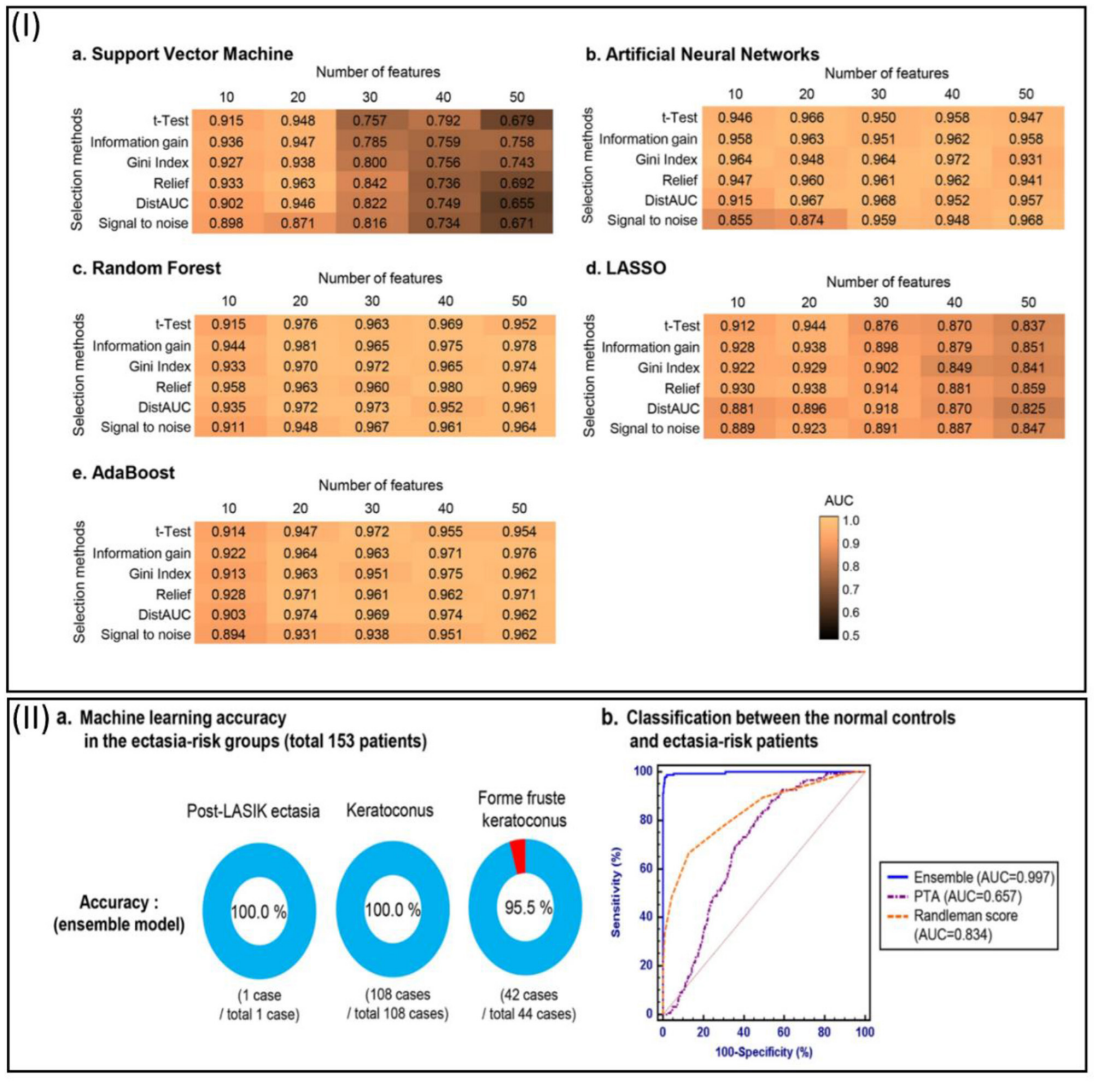
**(I)** To predict potential candidates for corneal refractive surgery, the heatmaps show the predictive performance (AUC) of feature selection and machine learning approaches. In this figure, the results of 10 fold cross-validation are indicated. a: Support vector machine. b: Artificial neural networks. c: Random forest. d: Least absolute shrinkage and selection operator (LASSO). AdaBoost. **(II)** Machine learning techniques are assessed for identifying ectasia-risk categories, including post-LASIK ectasia, keratoconus, and forme fruste keratoconus patients. a: Each group at risk of ectasia in an accuracy rate. b: ROC curves to classify the normal control (*N* = 9,556) and total ectasia-risk group (*N* = 153) (50).

Machine learning handles large amounts of data and accurately identifies cases (74). Recently, screening candidates for corneal refractive surgery has become increasingly crucial to prevent unwanted outcomes and improve decision-making. It seems that a definitive screening approach to address the likelihood of a misdiagnosis has not yet been developed. According to a study by Yoo et al., it is increasingly crucial to examine candidates with corneal refractive surgery to avoid problems (50). Five diverse methods were utilized to forecast potential candidates for surgery. Enhanced performance was achieved with an ensemble classifier. The model successfully reclassified a patient with post-operative ectasia as belonging to the ectasia-risk category. Refractive surgery can be performed with a secure and dependable clinical choice using automated machine learning analysis (Figure 3) (50).

### 4.2 AI for determination of correct effective drugs

The eye's complex physiological structures, diverse disease targets, limited drug delivery space, distinctive barriers, and intricate biomechanical processes pose significant challenges for treatment. Traditional screening approaches for formulation and manufacturing processes are inefficient for developing ocular formulations (75). Automated workflows and databases, alongside ANN implementation, have great potential to enhance treatment outcomes. These technologies enable rapid analysis of vast quantities of data, aiding in the development of innovative hypotheses and treatment strategies. Additionally, ANNs facilitate the forecasting of disease progression and pharmacological profiles. By leveraging these tools, significant advancements in treatments with better achievements are expected (76). The success of therapeutic interventions highly depends on subject selection, appropriate cell doses or medications (before and after surgery), and the number of administrations. Clinical pharmacology has a unique opportunity, regarding the availability of multidimensional data and the advancement of current methodologies for data analysis. Precision dosing with reinforcement learning is currently used for individualizing dosing regimens in patients with life-threatening diseases and in data science. It is referred to special issue as cutting-edge approaches to the collection, aggregation, and analysis of data, which can significantly contribute to characterizing drug-response variability at the individual level (77).

### 4.3 AI for detecting the correct area in patients undergoing corneal surgery

In previous studies, patients with advanced keratoconus have received cells for corneal stroma regeneration by creating a pocket in the corneal stromal tissue (57, 78). For this operation, determining an accurate target site with the correct diameter is very important to reduce possible complications. However, AI technology enhances surgical precision, decreases the need for human intervention, facilitates intraoperative decision-making, and boosts surgical safety. Nevertheless, there are still many obstacles to overcome before AI can be widely used in operating rooms (79).

AI can process OCT or confocal microscopy images to map corneal irregularities, such as stromal scars and limbal defects, and to identify optimal injection sites (80). Intraoperative live OCT combined with YOLO-based algorithms can track injected cell clusters to ensure correct placement and detect, for example, mesenchymal stem cells that have been misdirected during treatment (81).

**Figure 4:**
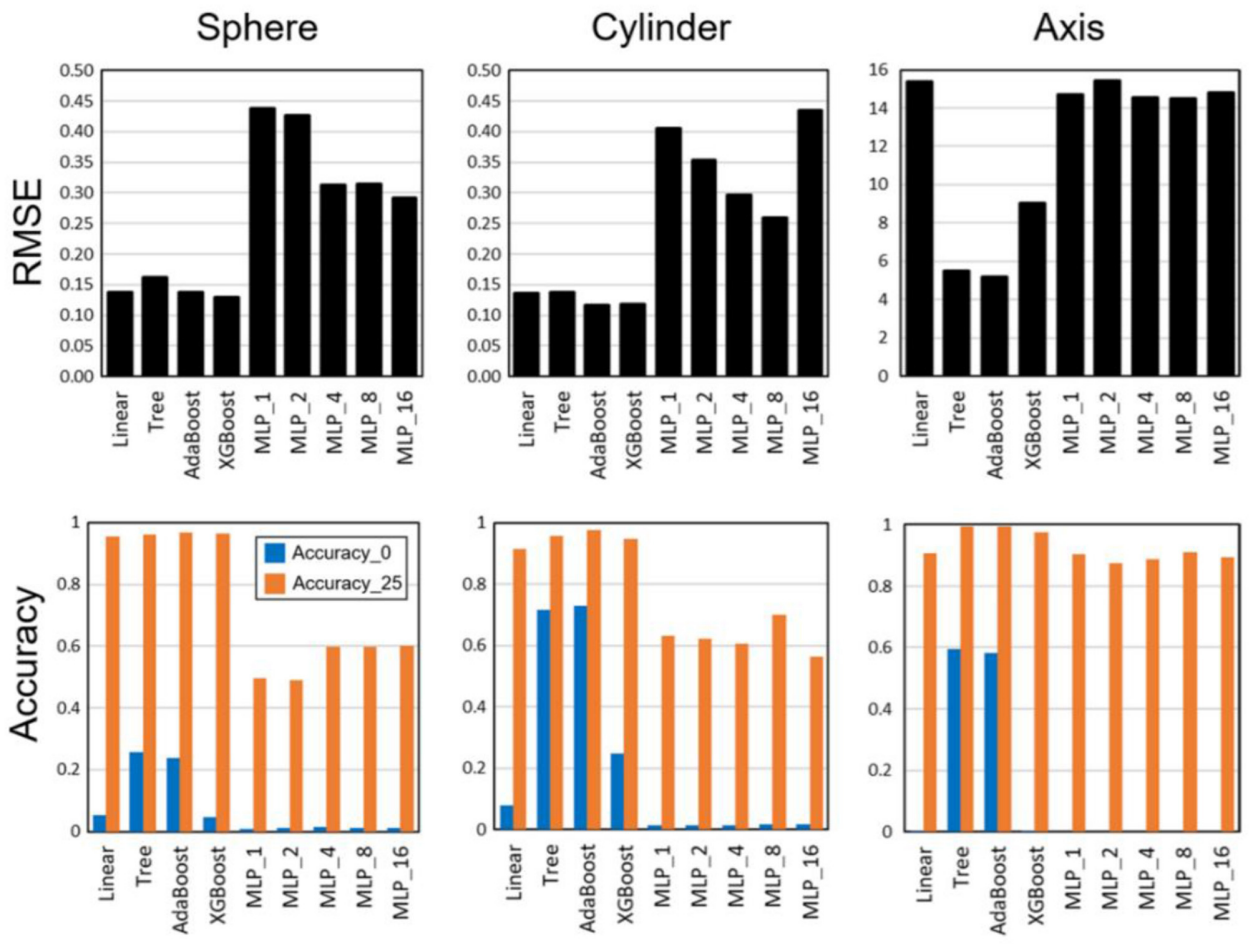
Root-mean-square errors (RMSEs) and accuracy results for multiple linear regression, decision tree, AdaBoost, XGBoost, and multi-layer perceptron (MLP) with hidden layers [Reprinted from reference (82)].

Ophthalmologists make a nomogram diagnosis by applying their specialized training and knowledge to pre-operative refractive data. For example, machine learning algorithms such as AdaBoost with the highest accuracy to predict sphere, cylinder, and astigmatism axis nomograms for accuracy in SMILE refractive surgery have been employed (Figure 4) (82). Notably, AI algorithms to pinpoint the location of damage and target sites accurately would be beneficial for clinical applications.

### 4.4 AI for determination of cell properties

The number of living cells after an injection or graft implantation is significant for predicting post-operative improvements in vision. As in an impressive study by Levine et al., an algorithm was developed to quantify activated dendritic cells (aDCs) using IVCM images (83). This study incorporated a total of 173 distinct images, each representing a unique individual. The estimation of the number of aDCs in the central cornea can be effectively determined through the algorithm based on automated machine learning. Forecasting cell counts could lead to greater advancements in corneal cell-based therapy. Finite element analysis combined with machine learning predicts the distribution of injected cells based on corneal stiffness and wound geometry (84). Huang et al. compared the application of five different machine learning algorithms, such as linear regression, support vector regression, decision tree regressor, RF regression, and extra tree regression (85). The RF regressor algorithm indicated the highest accuracy, at 80%, in predicting the presence or absence of cells within single droplets. Meanwhile, the extra tree regressor indicated the lowest mean error, of 12%, in anticipating the number of printed cells within multiple droplets. A combination of these models in a droplet monitoring system can be useful to determine the printed cell number under a live assessment throughout an inkjet-based bioprinting process.

Also, AI algorithms can process data from OCT, confocal microscopy, or slit-lamp imaging to track the integration of corneal cells following transplantation (84).

### 4.5 AI for detection of graft detachment

Diagnosing graft detachment to reduce complications after the surgery can be highly effective. According to a previous study, the efficacy of deep learning in the prediction of rebubbling after Descemet's membrane endothelial keratoplasty (DMEK) was evaluated (42). This investigation analyzed an equal number of eyes in both the rebubbling (RB) group and the non-RB group after DMEK. To categorize the RB group, a set of images was chosen randomly from the anterior segment OCT on day 5 after the operation. Training on a selection of nine deep neural network architectures, namely VGG16, VGG19, ResNet50, InceptionV3, InceptionResNetV2, Xception, DenseNet121, DenseNet169, and DenseNet201, was conducted. The VGG19 model demonstrated the highest area under the receiver operating characteristic curve among all the models.

Remarkably, these AI algorithms may have the potential for the detection of early graft detachment post-surgery.

### 4.6 AI for detection of infection

Effective treatment for the detection of infections and neovascularization in operated eyes is essential. Kuo et al. determined various deep learning algorithms that could detect bacterial keratitis from eye photographs (32). Five referral facilities were consecutively sampled to provide external eye pictures of suspected patients with infectious keratitis. The candidate deep learning frameworks—ResNet50, ResNeXt50, DenseNet121, SE-ResNet50, EfficientNets B0, B1, B2, and B3—were utilized to identify bacterial keratitis based on the receiver operating characteristic (ROC) curve. These models exhibited considerable potential as diagnostic tools for detecting bacterial keratitis.

### 4.7 AI for detection of edema and neovascularization

According to a prior investigation, AI algorithms have garnered significant attention in the field of macular disorders, specifically diabetic macular edema (DME) (86). In this study, the identification and quantification of different main OCT biomarkers in DME eyes in comparison to an algorithm to human expert manual analyses were considered. This may enable clinicians to consistently identify and measure OCT biomarkers related to DME, providing an objective approach to diagnosing and monitoring eyes affected by DME.

The application of three deep learning models, namely InceptionV3, ResNet50, and FLIMBA—for the automatic detection of corneal edema in second harmonic generation (SHG) images of the porcine cornea was assessed (87). SHG is a beneficial non-linear optical imaging tool to non-invasive identify, characterize, and monitor changes in the collagen structure of tissues under a contrast mechanism. Nevertheless, the analysis of SHG data is challenging, even for experienced histopathologists. This obstacle hinders the implementation of SHG-based diagnostic frameworks in clinical environments. The findings of this study were aimed at automating the determination of corneal hydration levels or corneal edema.

AI and learning techniques adjust the needle depth and angle in real time to prevent Descemet membrane rupture during the injection of corneal endothelial cells (CECs). This method may reduce the potential complications of cell injection, including edema (88).

Patients with corneal epithelial abnormalities benefit greatly from the detection of neovascularization and the expectation of reduced symptoms. In a previous study, OCT photographs were obtained at the beginning of the neovascular age-related macular degeneration process, and anti-VEGF injection doses were recorded following pro re nata (PRN) treatment (89). Data from the HARBOR research tracked patients who received PRN ranibizumab following three initial monthly injections for 2 years. The macular microstructure was described using quantitative spatiotemporal features obtained from automated segmentation of retinal layers and fluid-filled areas. Treatment categories were predicted and evaluated using RF classification and cross-validation, respectively. Anti-VEGF therapy requirements were suggested and evaluated with an OCT-based machine learning methodology. The results of this pilot study were a significant step toward the development of image-guided prediction of treatment intervals for the management of neovascular age-related macular degeneration. It is expected that this AI algorithm may be effective for the prediction of corneal neovascularization.

### 4.8 AI for the detection of changed stem cell morphology into target cell

In general, stem cells can differentiate into specific cells under physicochemical conditions. During differentiation, some phenotypic features of stem cells changed toward those of mature cells. These morphological changes can be recorded by microscopic observations. Moreover, observing functional markers of differentiated cells can be obtained using real-time PCR and immunobiological assay (90, 91). Meanwhile, detecting cells in the target site based on morphology features is valuable, especially for clinical studies. A novel AI system was developed to autonomously classify bone marrow cells and assess the potential clinical applications (92). Initially, a computerized analysis system known as Morphogo was employed to conduct comprehensive imaging of bone marrow smears. The findings of this preliminary investigation provided the Morphogo system as an automated tool for analyzing bone marrow cell differential counts. It appears that this AI algorithm might have the potential to identify and analyze cellular morphologies that hold potential advantages for corneal applications.

### 4.9 AI for detection of biomarker levels in subjects

One of the most important findings for the classification of diseases is the specific biomarker prediction. Recent technological advancements, particularly in the generation of extensive biological multi-omics datasets, have significantly broadened the scope of biomarker detection.

In a study by Chang et al. multiple machine learning algorithms were used to analyze transcripts from keratoconus patients, identifying characteristic gene combinations and their functional associations to enhance understanding of keratoconus pathogenesis (93). Machine learning models, including XGBoost, random forest, logistic regression, and SVM, identified a set of key genes associated with corneal ectasia. Notably, 15 genes—such as IL1R1, JUN, CYBB, CXCR4, KRT13, KRT14, S100A8, S100A9, and others—appeared across multiple models. Genes downregulated in keratoconus compared to the control group were involved in epidermal mechanical resistance (KRT14, KRT15) and inflammatory pathways (S100A8/A9, IL1R1, CYBB, JUN, and CXCR4). This study employed multiple machine learning algorithms to analyze transcripts from keratoconus patients, identifying characteristic gene combinations and their functional associations with the aim of enhancing the understanding of keratoconus pathogenesis. Machine learning models, including XGBoost, random forest, logistic regression, and SVM, identified a set of key genes related to corneal ectasia, with 15 notable genes consistently appearing in multiple models, such as IL1R1, JUN, CYBB, CXCR4, KRT13, KRT14, S100A8, and S100A9, among others. Genes downregulated in keratoconus compared to the control group played roles in epidermal mechanical resistance (KRT14, KRT15) and inflammatory pathways (S100A8/A9, IL1R1, CYBB, JUN, and CXCR4).

During the physical differentiation process, specific proteins are released from ADSCs when they are differentiated into various cell types, including corneal keratocytes (90). Prediction of the expression of many of them may be very significant for finding the vision recovery level in patients with keratoconus. It seems AI approaches may be effective to detect specific expressed markers in the specific tissues and cells. In a review article, evidence from the scientific literature regarding ocular imaging biomarkers is summarized, with a particular emphasis on the predominant role of biomarkers derived from OCT (94). The authors also note recent advancements in optical coherence tomography angiography (OCT-A) and experimental polarization-sensitive OCT (PS-OCT), which have revealed potentially informative novel biomarkers.

### 4.10 AI for detection of corneal cell junction

Tight junctions are crucial in the establishment of corneal homeostasis via epithelial and endothelial functions. Tight junctions are observed within the corneal epithelium, where a continuous pattern of zonula occludens (ZO)-1 can be identified at the apical cell borders (95, 96). Previous studies have shown that an increase in reactive oxygen species (ROS) levels leads to a decrease in tight junction proteins and compromises the epithelial barrier integrity (97–99). In freeze-fracture replica electron microscopy, the observed structures manifest as a cohesive network of fibrils, commonly referred to as tight junction strands. Tight junction strands serve as molecular zippers, effectively establishing a physical barrier to impede the paracellular diffusion of molecules. The morphology of the tight junction strand network exhibits significant variation across different tissues (100). To confirm newly discovered molecules and localize them to the tight junction, experiments like immunofluorescence investigations can be performed. Understanding tight junction signals between healthy or diseased corneal cells would help to determine the success rate of the treatment.

### 4.11 AI for detection of corneal curvature

Intra-corneal ring implantation has recently emerged as a viable alternative to corneal transplantation for keratoconus treatment (101–103). Predicting outcomes after this procedure is important for clinicians to select the most appropriate pre-operative variables. A novel machine learning-based approach can be employed to forecast the visual improvement of patients with keratoconus after ring implantation. The measurement of corneal curvature and astigmatism can be used to determine the vision gain (104). In another study, a developed AI model used multiple tomographic parameters to evaluate local against global keratoconus progression (105). Collectively, the AI models recognized the eyes with changes in parameters like an increase in maximum anterior curvature (Kmax) and others related to disease progress. These models can be optimized to predict the outcome of cell therapy and tissue engineering processes. Machine learning models can detect subtle signs of immune rejection or cell death prior to the onset of clinical symptoms, enabling early diagnosis of transplant rejection in keratoconus (106).

## 5 Forecasting recovery time based on satisfied signs using AI

The capacity to predict the duration required in a surgical procedure for both patients and medical practitioners is desirable. Reliable clinical judgment can be obtained through automated machine learning applied to pre-operative data. This cutting-edge technology for lowering the risk of problems in patients is significant (107). For example, AI techniques such as machine learning and deep learning have found a suitable application in anesthesiology. This is due to the substantial volume of data produced during perioperative surgery and anesthesia management (108). AI platforms may be useful for predicting patient repair with epithelial abnormalities, keratoconus, edema, or even blindness. For example, AI can be used to predict ocular hypertension following Descemet membrane endothelial keratoplasty (DMEK) (109).

## 6 Conclusion and future perspectives

Cell therapy is an emerging medical field that utilizes living cells to address a range of diseases and problems. AI has the potential to speed up the development of cell therapy by supplying insights, forecasts, and optimizations at various stages. For example, AI can help identify new targets for cell treatment by examining extensive genomic, transcriptomic, and proteomic data, and patient-specific details. Using biological and clinical factors, AI can assist in determining the most relevant and achievable targets. Computer algorithms can assist in optimizing the design of cell therapy payloads, including genetic modifications, receptors, and signaling pathways that ensure a cell's functionality and selectivity. AI-based approaches can explore and utilize the extensive design possibilities offered by these modalities, saving time and reducing experimentation costs. Despite ongoing challenges—such as limited and variable data quality, model interpretability and validation hurdles, and ethical considerations—effective use of AI for cell therapy requires robust cross-disciplinary and cross-sector collaboration and dialogue (18, 110, 111). One of the most significant challenges in applying AI is algorithmic bias. Intrinsic biases can emerge during development and clinical deployment, leading to inaccuracies and variability in model outputs. Pinpointing the sources of bias—whether from data sampling, labeling, feature selection, model design, or deployment context—is difficult. If unaddressed, biased AI can drive non-standard clinical decisions and exacerbate healthcare disparities (112, 113). Moreover, the absence of comprehensive and sufficient regulations for overseeing AI development and usage, along with concerns about data safety and transparency, are significant challenges that require special attention (114, 115). Optimized treatment scenarios using AI may be more realistic by encompassing a single treatment option or determining the most effective combination of treatments. For instance, it would be attractive for treatments to be accompanied by an accurately prescribed dosage, which plays a crucial role in evaluating individual therapeutic methods. Employing AI algorithms for personalized corneal cell-based therapies can effectively aid in administering sufficient cell numbers to patients. The exact cell injection location in keratoconic corneas may be detected by AI algorithms. Therefore, these patients might receive sufficient cells at optimized location for improving vision.

Moreover, it should be noted that AI might be reliable in the healthcare field for cell therapies using supportive data. AI is needed to combine with clinical and laboratory data for managing some corneal diseases, such as keratoconus. Detection errors would be minimized by connecting AI with current techniques, such as clinical images obtained from OCT and molecular evaluations. Insufficient investigation regarding the real-world performance, generalizability, and interpretability of AI systems needs more attention in future studies (116). Resolving issues by data sharing, data annotation, and other interconnected challenges will effectively expedite the advancement of more resilient AI products. Ultimately, one of the important priorities that can foster special attention to the role of AI in ophthalmology research and eye cell therapy is multi-center validation of this emerging technology. When integrated with clinical workflows, it ensures that this innovative medical approach is reliable and effective.

An AI language model was utilized to enhance the clarity and grammar of this manuscript.
