# Correction: AI-driven strategies for advancing corneal cell therapy: a promising frontier

**DOI:** 10.3389/fmed.2025.1722730

**Published:** 2025-11-11

**Authors:** Mahsa Fallah Tafti, Masoud Khorrami-Nejad, Masoud Arabfard, Mohsen Ghiasi, Fatemeh Afkhamizadeh, Khosrow Jadidi, Hossein Aghamollaei

**Affiliations:** 1 Vision Health Research Center, Semnan University of Medical Science, Semnan, Iran; 2 School of Rehabilitation, Tehran University of Medical Sciences, Tehran, Iran; 3 Optical Techniques Department, College of Health and Medical Techniques, Al-Mustaqbal University, Babylon, Iraq; 4 Artificial Intelligence in Health Research Center, Biomedicine Technologies Institute, Baqiyatallah University of Medical Sciences, Tehran, Iran; 5 Cardiovascular Research Center, Rajaie Cardiovascular Institute, Tehran, Iran; 6 Chemical Injuries Research Center, Systems Biology and Poisonings Institute, Baqiyatallah University of Medical Sciences, Tehran, Iran

**Keywords:** artificial intelligence, cornea, cell therapy, regenerative medicine, personalized medicine

Affiliation “Chemical Injuries Research Center, Systems Biology and Poisonings Institute, Baqiyatallah University of Medical Sciences, Tehran, Iran” for corresponding author, Hossein Aghamollaei, was erroneously given as “Artificial Intelligence in Health Research Center, Baqiyatallah University of Medical Sciences, Tehran, Iran”

Affiliation “Artificial Intelligence in Health Research Center, Biomedicine Technologies Institute, Baqiyatallah University of Medical Sciences, Tehran, Iran” was erroneously given as “Artificial Intelligence in Health Research Center, Baqiyatallah University of Medical Sciences, Tehran, Iran”

The original version of this article has been updated.

